# Satisfaction with primary care and mental health care among individuals with severe mental illness in a rural area: a seven-year follow-up study of a clinical cohort

**DOI:** 10.1186/s13033-016-0064-8

**Published:** 2016-04-12

**Authors:** Torleif Ruud, Trond F. Aarre, Berit Boeskov, Per Stå le Husevåg, Rigmor Klepp, Synnøve Alet Kristiansen, Jorunn Sandvik

**Affiliations:** Division of Mental Health Services, Akershus University Hospital, 1478 Lørenskog, Norway; Institute of Clinical Medicine, University of Oslo, Oslo, Norway; Nordfjord Psychiatric Center, Nordfjordeid, Norway; Child and Adolescent Psychiatric Department, Center for Eating Disorders, Region Sjælland, Denmark; Child Welfare, Gloppen, Norway

**Keywords:** Severe mental illness, Patient satisfaction, Follow-up, Community mental health center, Primary care, General practitioners

## Abstract

**Background:**

Most studies of services for people with severe mental illness have been performed in cities. Our 7-year follow-up study aimed to investigate clinical course and satisfaction with services among individuals with severe mental illness who received community mental health services in a rural area. The services were provided by primary care and a community mental health center (CMHC), which worked in close collaboration and emphasized individually tailored case management, relationship-building and continuity of care.

**Methods:**

All 57 patients with severe mental illness who were seen by the CMHC in 1992–1993 and were still alive in 1999 were asked to participate. Retrospective ratings were performed for the first month of contact in 1992–1993 based on patient records and detailed notes. A semi-structured interview was conducted in 1999–2000 with the 40 patients (70.2 %) who gave written consent to participate in the study. DSM-IV diagnoses were made using OPCRIT. The retrospective baseline ratings and the follow-up interview included assessments of symptoms and functioning using the following instruments: the Brief Psychiatric Rating Scale Expanded version 4 (BPRS-E), the Health of the Nation Outcome Scales (HoNOS), the Global Assessment of Functioning Scale (split version), and the Practical and Social Functioning Scale (PSF).

**Results:**

The ratings revealed improvements in psychiatric problems and functioning. Patients with schizophrenia spectrum disorders improved primarily in psychotic symptoms, while patients with severe affective disorders improved primarily in affective symptoms. Large variations in the use of primary care and mental health services were observed, with more intensive specialized mental health services for individuals with schizophrenia spectrum disorders than severe affective disorders. Overall, the patients were satisfied with the provided services. They were most satisfied with GPs and more satisfied with local outpatient and inpatient services than with hospital inpatient services and medication.

**Conclusions:**

Patients with severe mental illness in a rural area value local services that emphasize relationships and close collaborations among the CMHC, GPs and primary health and social care. Even in an area with a fairly well-staffed CMHC, the highest patient satisfaction was reported for GPs, indicating the potential key role of GPs for this patient group.

## Background

This paper reports patient satisfaction and clinical course from a 7-year follow-up study of a cohort of service users with severe mental illness (SMI) who received collaborating services from primary care (including general practitioners, GPs) and a community mental health center (CMHC) in a rural area on the west coast of Norway. As large-scale development of community mental health care is still in an early phase in many countries [1, 2], experiences related to the close collaboration of primary care services and a pioneering CMHC in Norway 1992–2000 may be of interest for others who are currently developing local mental health services in rural areas.

Most studies of community mental health services for individuals with SMI have been performed in cities. Studies of shared care by collaboration of primary care including GPs and specialized mental health care have been performed primarily for patients with depression, and few studies are available concerning the experience of combined treatment from primary care and CMHC for individuals with SMI [3, 4]. This type of combined treatment may be the most common type of services for this target group, especially in rural areas.

According to patients and health professionals, GPs are important for patients with SMI [5]. The engagement of GPs in the treatment of individuals with SMI depends on their interest in mental health and other factors like the support available from collaborating mental health services [6]. Good communication between services is important for the quality of the services [7]. In addition, mental health nurses, primary care mental health teams and social services are important collaborators for the GP and the CMHC.

In Norway, the development of CMHCs with outpatient clinics and inpatient wards was one of the main strategies in a national plan for mental health 1999–2008 [8]. Much of the country consists of rural areas, and during the ten-year plan, resources were increased for CMHCs and for primary care in the municipalities. However, a report on all of the CMHCs in the country in 2013 revealed that there is still great variation in the available resources and the degree of implementation of the different types of community mental health services [9]. In rural areas the number of psychiatrists is one of the critical factors for capacity and quality of outpatient and local inpatient treatment at CMHCs for persons with SMI. In two recent national surveys of GPs’ assessment of the local CMHC showed that many CMHCs still have great potential for the improvement of their services [10, 11].

Countries and areas that are developing community mental health services based on collaboration by primary care and CMHCs may take into account the experiences from similar processes during the last three decades in Norway. Studies describing the content of shared care and patient satisfaction with this care over time may be especially valuable. However, recent Norwegian studies have been primarily cross-sectional studies describing the types of services provided, but not following the patients over time and reporting course and outcome. One 6-year study followed a group of patients who were receiving long-term inpatient care at the time of the baseline evaluation [12, 13], but this study reported limited information concerning the content of the treatment provided in the communities after discharge from institutions, and it had no measures of patient satisfaction.

### The local area and the collaborating services

Nordfjord is a rural area surrounding a fjord on the west coast of Norway. There are six small municipalities in the area, with a total population of 30,000. An epidemiological study found a lower prevalence of affective disorders but a similar prevalence of non-affective psychoses in this rural county in comparison to the Norwegian capital Oslo [14]. The CMHC is located in the middle of the area, with a 1 hour drive to most of the municipality centers. At the time of the follow-up study, 20 GPs were in the area and each municipality provided primary care by mental health workers, social services and supported housing for persons with SMI. In the small municipalities, the GPs and other primary care workers had a good knowledge of the population and the local community. High stability and low turnover among GPs and health workers contributed to high continuity of care and long-term personal contact with patients. Most GPs were highly engaged in serving patients with SMI and other mental disorders, and the GPs expressed that the close collaboration with the CMHC encouraged them to take more active responsibility for these patients.

Nordfjord CMHC was one of the first CMHCs in Norway to provide the full range of outpatient, day patient, mobile and inpatient services, as intended in national plans. The CMHC became fully operational in 1992 and has subsequently provided treatment in close collaboration with GPs and other municipal primary health and social services in the catchment area. At the time of the follow-up study, the CMHC had an outpatient clinic with seven clinicians, a mobile rehabilitation team with a staff of four individuals, a day unit with a staff of three individuals, and two nine-bed inpatient wards with a staff-patient ratio 1.5:1.0. One of these wards was for patients with SMI, and the other unit was for all other patient groups.

Four psychiatrists were available at the CMHC. The staffing was considered to be fairly good for a small CMHC, and the CMHC has been rated highest in quality in two national surveys of GPs’ ratings of the local CMHC [10, 11]. This indicates that the quality of local mental health care and collaboration in our area was considered to be fairly good.

Most of the needs for psychiatric inpatient services and all of the needs for psychiatric outpatient services in the local area were covered by the CMHC. The CMHC also collaborated closely with the mental health clinic at the county central hospital 2–3 h from Nordfjord, which had acute and closed inpatient psychiatric wards. On average, four inpatients from Nordfjord were present in these wards. Involuntary admissions could only be processed at the mental health clinic at the county central hospital, but involuntary treatment could be transferred to the CMHC and continued there.

A major component of the care available from the CMHC for people with SMI was clinical case management by the staff of the mobile rehabilitation team, as well as of outpatient and inpatients units. Building and maintaining relationships and alliances with the patients and continuity of care were emphasized. Primary care or CMHC staff met SMI patients weekly or more often in their homes, in the community or in localities of the services. Important treatment components included help and support in everyday living, meaningful activities, training in practical and social skills, medication, supportive psychotherapy and meeting the family [15]. During the years of the follow-up study, clinical guidelines and evidence-based treatments were emerging and began to influence clinical practice, but specific models and fidelity measures were still scarce and not implemented in the services.

The shared care provided by the primary care and mental health care services was implemented partly as joint service delivery and partly by close coordination of the services provided by each agency. The psychiatrists and other clinicians from the CMHC spent 1 day every week working in the municipalities with the GPs and primary care. This service included joint consultations, home visits, family sessions and supervision. The close collaboration led to good working relationships based on mutual knowledge and respect and increased the overall competence of the shared care. The coordination of the total services for each patient was accomplished in meetings every 6–8 weeks for all professionals involved with the patient, with one case manager from the primary care or the CMHC as coordinator. The CMHC also had close contact with the psychiatric inpatient department at the central hospital, facilitating early discharge to the community or transfer to the CMHC inpatient ward.

In 1996–1998, more than thirty health professionals from the CMHC and municipality primary care services participated in a comprehensive 2-year local training program in community mental health care for people with SMI. This program was arranged in collaboration with the Center for Psychotherapy and Psychosocial Rehabilitation of Psychoses (SEPREP), which is a national network of clinicians, service users and caregivers. The participants received supervision every second week in joint supervision groups for health workers from both the CMHC and primary care, attended 1 day of lectures every month, and met in groups to discuss clinical literature every month [16]. The training was part-time and completed in parallel to clinical work and aimed to increase the competency of clinical practice during the training. From 1999, SEPREP was commissioned by the government to build a national program with local training programs based on this model. This national program has been widely disseminated and is still running with strong impacts throughout Norway.

### Research questions

The aims of this paper are to: (1) Describe the clinical course of patients with SMI during the 7-year follow-up period and (2) Report patient satisfaction with service components and with the collaboration of services.

## Methods

### Design

This study is a 7-year follow-up study of a 2-year clinical cohort of patients with severe mental illness. The baseline assessment was performed retrospectively but before the follow-up interview.

### Material

The 2-year clinical cohort was defined as the individuals with SMI (schizophrenia spectrum disorders and severe affective disorders) who had been outpatients and/or inpatients at the CMHC in 1992–1993. These years were the first 2 years that the CMHC was in full operation, and we identified 64 patients who fulfilled the criteria. All of the 57 patients who were still alive in 1999 were contacted by letter and invited to take part in the follow-up study, and 40 patients (70.2 %) gave written consent. No significant differences in gender, age or retrospective baseline ratings of the severity of psychiatric problems were observed between the 40 patients who gave consent and the 17 patients who did not give consent.

### Instruments and variables

DSM-IV diagnoses were made using Operational Checklist for Psychotic Symptoms, version 3.4 (OPCRIT) with algorithms based on 90 criteria from the WHO Schedule for Clinical Assessment in Neuropsychiatry (WHO-SCAN) [17]. We used all available information to answer the 90 diagnostic criteria for psychotic and affective symptoms, and used the OPCRIT software to get a lifetime diagnosis. The type and severity of psychiatric symptoms were rated using the Brief Psychiatric Rating Scale, Expanded Version 4.0 (BPRS-E) [18]. The type and severity of recent problems in major problem areas in relation to SMI was rated using the Health of the Nation Outcome Scales (HoNOS) developed to cover these areas listed in Table 1 [19]. Functioning was measured using the Global Assessment of Functioning Scale (GAF) (split version, with separate scoring of symptoms and function) [20] and the Rating Scale for Practical and Social Functioning (PSF) [21].

**Table 1:** Paired sample *t*-test of clinical characteristics at baseline and follow-up (N = 40)

Variables	Baseline^a^	Follow-up	Improvement		
	Mean (SD)	Mean (SD)	Mean (SD)	CI 95 %	p
Brief Psychiatric Rating Scale (BPRS-E)^b^					
Positive symptoms	2.45 (1.13)	1.90 (1.01)	0.55 (1.10)	(0.20–0.90)	0.003
Negative symptoms	2.08 (1.01)	1.58 (0.84)	0.50 (0.95)	(0.19–0.81)	0.002
Depression anxiety	2.48 (1.11)	1.89 (0.83)	0.59 (1.37)	(0.15–1.03)	0.010
Manic hostility	1.97 (1.07)	1.72 (1.10)	0.25 (0.94)	(−0.05–0.55)	0.104
BPRS mean total score	2.23 (0.68)	1.82 (0.80)	0.41 (0.70)	(0.18–0.63)	0.001
Health of the Nation Rating Scales (HoNOS)^b^					
1. Overactive, aggressive or agitated behavior	1.13 (1.28)	0.37 (0.68)	0.76 (1.05)	(0.42–1.11)	<0.001
2. Non-accidental self-injury	0.47 (0.86)	0.16 (0.37)	0.32 (0.93)	(0.01–0.62)	0.044
3. Problem drinking or drug-taking	0.24 (0.68)	0.08 (0.49)	0.16 (0.50)	(−0.01–0.33)	0.057
4. Cognitive problems	1.13 (1.38)	0.63 (0.94)	0.50 (1.13)	(0.13–0.87)	0.010
5. Physical illness or disability	0.57 (0.96)	0.89 (1.18)	–0.32 (1.00)	(−0.66–0.01)	0.057
6. Hallucinations and delusions	2.00 (1.41)	1.24 (1.34)	0.76 (1.64)	(0.23–1.30)	0.007
7. Depressed mood	1.35 (1.41)	0.60 (0.94)	0.74 (1.62)	(0.17–1.30)	0.012
8. Other mental and behavior problems	0.97 (1.19)	0.84 (1.19)	0.14 (1.53)	(−0.38–0.65)	0.595
9. Problems with relationships	2.11 (1.16)	1.58 (1.13)	0.53 (1.08)	(0.17–0.88)	0.005
10. Problems in activities of daily living	1.71 (1.45)	1.53 (1.22)	0.18 (1.45)	(−0.29–0.66)	0.438
11. Problems with living conditions	0.92 (1.28)	0.45 (0.76)	0.47 (1.43)	(0.00–0.94)	0.048
12. Problems with occupation and activities	1.05 (1.14)	0.97 (1.33)	0.08 (1.53)	(−0.42–0.58)	0.752
Practical and Social Functioning Scale (PSF)^b^					
A. Care for health	4.62 (3.54)	8.18 (2.51)	3.56 (3.43)	(2.45–4.68)	<0.001
B. Self-care/clothes	6.77 (3.48)	8.18 (2.51)	1.41 (2.91)	(0.47–2.35)	0.004
C. Meals and food	6.33 (3.30)	7.79 (2.93)	1.46 (3.55)	(0.31–2.61)	0.014
D. Care for belongings	6.82 (3.42)	8.28 (2.08)	1.46 (2.94)	(0.51–2.41)	0.004
E. Managing finances	7.10 (3.14)	8.13 (3.14)	1.03 (3.06)	(0.03–2.02)	0.043
F. Use of transportation	5.05 (3.33)	6.18 (3.49)	1.13 (2.89)	(0.19–2.07)	0.020
G. Social contact	5.74 (3.49)	6.13 (3.21)	0.38 (3.13)	(−0.63–1.40)	0.448
H. Conversations	5.82 (3.08)	8.38 (2.86)	2.56 (2.56)	(1.73–3.39)	<0.001
I. Ability to work	5.85 (3.11)	7.38 (3.23)	1.54 (3.49)	(0.41–2.67)	0.009
J. Leisure activities	4.31 (3.02)	5.51 (2.68)	1.21 (3.11)	(0.20–2-21)	0.020
Global Assessment Scale (Split GAF)^b^					
GAF symptoms	32 (13)	45 (20)	13 (19)	(7–20)	<0.001
GAF functioning	36 (11)	47 (18)	11 (18)	(517)	<0.001

^a^Rated retrospectively based on patient records prior to the follow-up study interview

^b^Scores for the BPRS-E range from 1 (none) to 7 (extremely severe). Scores for the HoNOS range from 0 (no problem) to 4 (severe to very severe problem). Scores for the PSF range from 0 to 10 (sum of five items of functioning rated 0–2, where 0 is “not true”, 1 is “partly true or true part of the time”, and 2 is “true or true the whole time”). Scores for the GAF range from 1 (as severe as possible) to 100 (as well as possible)

A questionnaire measuring patient satisfaction with each of the local service components was designed for the study as such specific components were not covered in established instruments for measuring patient satisfaction with services. We also included questions concerning the amount of each service component received by each patient.

The three psychiatrists doing the follow-up interviews discussed the rating scales thoroughly when preparing for the study. Each psychiatrist did their part of the 40 interviews, and for 18 of the interviews it was possible for one of the other psychiatrist to be present as a listener and do independent ratings on BPRS-E, GAF and PSF so that we could calculate inter-rater reliability (ICC) for the ratings on these instruments [22]. The ICC values were 0.85–0.94 for the four subscales of the BPRS-E, 0.92 and 0.91 for the GAF scales on symptoms and functioning, respectively, and 0.73–0.94 for the ten PSF subscales.

### Data collection and data analysis

The patients were retrospectively rated for psychopathology and level of functioning for the first month (the index month) of contact in 1992–1993, using all available information in written records and detailed notes that were made by the staff who worked with the patient. These ratings were made before the patients were contacted for a follow-up interview. The three mental health nurses in the project group conducted the retrospective baseline ratings based on patient records and detailed notes from any inpatient stays at that time, with support from the three psychiatrist in rating BPRS-E and HoNOS. The three psychiatrists conducted some months later the ratings in the follow-up interview without having access to the retrospective baseline ratings done earlier. None of the raters were blind to the aims of the study to learn about the clinical course and experiences of the patients.

In the follow-up interview in 1999–2000, which was 7 years after the index month, the patients were assessed using the same rating scales for psychopathology and the level of functioning that were used in the retrospective baseline ratings. They also answered the questions on patient satisfaction with service components. Going through the list of the service components listed in Table 2, the patient was first asked if he had received this type of service during the 7 years. If so, three additional questions were asked, reading the answers to choose among for each question: How much did you have of this during the years (from 0 = nothing to 4 = very much or all the time/most of the time)? How important has this been to you (from 1 = not important to 4 = very important)? How satisfied have you been with this type of service (1 = very dissatisfied, 2 = quite dissatisfied, 3 = mixed, 4 = quite satisfied, 5 = very satisfied)? One patient with extreme withdrawal and one patient with severe cognitive dysfunction were not able to answer all of the questions on patient satisfaction.

**Table 2:** Contact (%) and satisfaction with services reported by patients (N = 38)^a^

	Proportion of patients reporting contact with service component	Satisfaction^b^ with service component
	%	Mean (SD)
Primary care		
GP	69	4.44 (0.58)
Sheltered/supported work	41	4.15 (0.90)
Social assistant	30	3.90 (1.29)
Day center in municipality	27	3.89 (1.17)
Primary care nurse	61	3.65 (0.93)
Social services	46	3.43 (1.22)
Psychopharmacological treatment	97	3.41 (1.08)
Specialized mental health care		
Outreach from CMHC	24	4.25 (0.71)
Inpatient stay at CMHC	89	4.03 (0.95)
Case management	44	4.00 (0.93)
Psychiatrist	50	4.00 (0.94)
Day-patient at CMHC	26	3.78 (0.67)
Individual therapy	50	3.59 (1.12)
Family sessions	32	3.27 (1.01)
Inpatient stay at central hospital	60	3.10 (1.22)
Group therapy	29	2.60 (1.17)
Family and friends		
Support from family	80	4.07 (0.86)
Support from friends	52	3.89 (0.76)
Other aspects of services		
Satisfaction with coordination	–	3.67 (0.80)
Satisfaction with MHC family contact	–	3.58 (1.20)
Satisfaction with information	–	3.49 (1.22)
Satisfaction with own influence	–	3.37 (1.11)

^a^Two patients were unable to answer due to extreme withdrawal or severe cognitive dysfunction

^b^Scale for satisfaction ranged from 1 = very dissatisfied through 3 = mixed to 5 = very satisfied

Data analyses were performed using SPSS for Windows version 18 and included descriptive statistics and analyses of the significance of differences that employed the Chi square test and the Student’s *t* test.

## Results

### The clinical course of patients

Table 1 shows the clinical characteristics of the sample at baseline and follow-up. The group with schizophrenia spectrum disorders included 17 patients with schizophrenia and 6 patients with schizoaffective disorder. The group with severe affective disorders included 11 patients with bipolar disorder and 6 patients with depression with psychotic features. All the patients had a well-established long term severe mental illness with one or more illness episodes at the index month in 1992–1993, and none had a recent onset first episode. The mean time since the start of the severe illness was 14 years (SD 14 years).

There were 10 women and 13 men with schizophrenia and 8 women and 9 men with severe affective disorders. Distribution on age groups was fairly equal in the two patient groups. Two patients with schizophrenia and seven with severe affective disorder were living with a spouse or partner at follow-up. There was an increase in the number with disability pension from 14 to 20 of those with schizophrenia and from 6 to 10 of those with affective disorder. Six with affective disorder were in paid work at follow-up compared to four at the index month, while none with schizophrenia were in paid work at follow-up compared to one at the index month.

Significant improvements of psychiatric problems and functioning were observed for several subscales, as shown in Table 1. The severity of psychiatric symptoms was reduced during the follow-up period for both groups, but these changes had different patterns. Patients with schizophrenia spectrum disorders improved significantly with respect to positive and negative symptoms on the BPRS and in the BPRS mean total score, with no significant changes in the subscales on affective symptoms. The patients with severe affective disorders showed the opposite pattern, with a significant reduction on the depression subscale but no significant changes in positive and negative symptoms, which were fairly low at baseline. The ratings on the GAF symptom subscale improved significantly for both groups. Both groups also experienced significant improvements in practical and social functioning, as measured using the PSF.

### Care and satisfaction with care

**Fig. 1:**
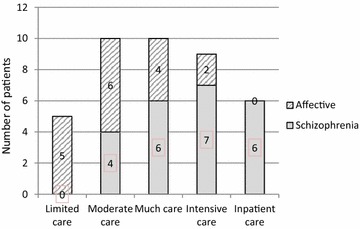
Number of patients with severe affective disorders and schizophrenia spectrum disorders receiving various degrees of intensity of care during the 7-year follow-up (N = 40)

Figure 1 shows the distribution of intensity of care for the total sample and for the two subsamples with schizophrenia spectrum disorders and severe affective disorders. A clear difference was observed between the two groups, with a higher intensity of care for the patients with schizophrenia spectrum disorders than for those with affective disorders. The six individuals who required inpatient care during most or all of the 7 years all had schizophrenia spectrum disorders, and the five patients who needed limited care all had affective disorders.

Table 2 shows the proportion of patients who reported contact with various service components within primary care and mental healthcare during the 7 years of follow-up. The pattern was largely the same for both groups of patients. Nearly all of the patients used medication, and most of the patients had been inpatients at the CMHC. Most patients reported contact with GPs and primary care nurses, but more variation was observed in the amount of contact with psychiatrists, social services and other types of services. Most patients also reported support from family, and half of the patients reported support from friends.

Table 2 also shows patient satisfaction with the services with which the patients had been in contact. Within each service level, the service components are ranked according to the level of satisfaction. The patients were generally satisfied with the services and were most satisfied with GPs. The patients were also satisfied with outreach from the CMHC, sheltered work, support from family, inpatient stays at the CMHC and contact with the case manager and psychiatrist at the CMHC. All of these services were on average rated 4 (satisfied) or above. The patients were somewhat less satisfied with inpatient stays at the county central hospital, family sessions, their own influence on the treatment, medication, information and group therapy.

## Discussion

Our 7-year follow-up study aimed to investigate clinical course and satisfaction with services among persons with severe mental illness who received community mental health services in a rural area. The services were provided by GPS and other primary care services and a CMHC. These services worked in close collaboration and emphasized individually tailored case management, relationship-building and continuity of care. The follow-up study showed improvements in psychiatric problems and functioning. Patients with schizophrenia spectrum disorders improved primarily in psychotic symptoms, while patients with severe affective disorders improved primarily in affective symptoms. Large variations in the use of primary care and mental health services were observed, with more intensive specialized mental health services for individuals with schizophrenia spectrum disorders than individuals with severe affective disorders. Overall, the patients were satisfied with the provided services. The patients were most satisfied with GPs and more satisfied with local outpatient and inpatient services than with hospital inpatient services and medication.

The 23 patients with schizophrenia spectrum disorders and 17 with severe affective disorders were 0.09 and 0.07 % of the adult population in the CMHC catchment area, respectively. An epidemiological study in our county found a life time prevalence of 0.4 % for non-affective psychoses and 0.2 % for bipolar disorder [14]. Taking into account some uncertainty of such estimates due to low prevalence of the disorders, our cohort seems to have a fairly representative balance of the two patient groups.

### The clinical course

There were improvements in both psychiatric problems and functioning during the 7 years from the index month to follow-up. In this observational study with retrospective rating for baseline we do not claim that the improvement is due to treatment effect. But part of the improvement may be due to the services given, as the results are in line with randomized controlled trials and other studies on integrated care for patients with schizophrenia [23]. A 6-year follow-up study of case management for patients with SMI in Sweden at the same time found an improvement in self-reported psychiatric symptoms, psychosocial functioning, social networks and quality of life [24]. But the Swedish study did not measure patient satisfaction with the services.

In our study there were large variations in the use of primary care and mental health services. Patients with schizophrenia spectrum disorders needed more intensive psychiatric outpatient services and more psychiatric inpatient services than patients with severe affective disorders, which may be expected from the severity of symptoms and functional impairment.

In our area with small municipalities and good stability and continuity of staffing for most services, the primary care and the CMHC were usually able to continue treatment and contact for as long as the user wanted and needed. The amount of contact and types of services used were thus largely based on the patient’s choice. In our treatment approach, we emphasized treatment that was individually adjusted according to changing needs through different phases of the clinical course. This makes it difficult to know whether more services are always better than less. We have found no established methods for measuring the level of individual adjustment of treatment, which could be useful in health services research.

### Patient satisfaction with care

Overall, the patients were satisfied with the provided services. The patients were most satisfied with GPs and were more satisfied with local outpatient and inpatient services than with hospital inpatient services and medication. The highest satisfaction was reported for services that require personal contact with the same person over time.

We are not aware of any similar follow-up study of SMI patients measuring satisfaction with primary care including GPs and specialized mental health care in rural areas. The Swedish 6 years follow-up study on case management reported a reduction in the use of mental health services after inclusion in case management, but it did not measure patient satisfaction with the services [24].

The patients were more satisfied with GPs than with any other service. This finding demonstrates the importance of the GP for individuals with SMI, even in an area in which the CMHC was rated highest in quality by GPs in national surveys. The continuity of a personal relationships between the patients and the GP may be one aspect contributing to this, as this has been shown to be one of the key factors in satisfaction of SMI patients with the GP [25]. GPs are in the local community, they know the local context and local services, and they may refer to local and specialized health service. GPs are providing both somatic and mental health care, as well as coordinating various services that may be needed.

The patients were also more pleased with inpatient stays at the CMHC than with inpatient stays at the county central hospital. This difference may be partially due to the different roles of these inpatient wards, as involuntary admissions were done at the central hospital. There are almost no studies done on inpatient treatment in CMHC, but a study of CMHC inpatient units in the UK also showed higher patient satisfaction at local inpatient units [26].

The patients were less satisfied with their own influence on the care and with the coordination of the services. This was an unexpected finding, as we aimed to put emphasis on patient involvement and coordination.

### Strengths and limitations of the study

Our sample consisted of most of the patients from the total 2-year cohort and may be considered to be a representative sample of the patients with severe mental illness during the inclusion period. However, the sample was fairly small and gave limited possibilities for sophisticated statistical analyses. In a small rural area, it is not possible to get the large samples that are more common in studies in urban areas. Our study also has other limitations. The rating of the clinical state at baseline was done retrospectively after 7 years. Thus, our knowledge of the later clinical course and the present state of some of the patients may have influenced our ratings despite our efforts to avoid such bias. The ratings at follow-up were done by psychiatrists who had been in charge of some of the treatment during the 7 years, and not by independent researchers. This may have given some unknown bias due to wishes to see positive changes in the patients. The retrospective ratings were based on the first period of contact with the patient in 1992–1993, and their condition were probably more severe immediately following referral compared with later. Regression to the mean during the follow-up period obviously contributes to the differences between the baseline and follow-up ratings, and we cannot prove what part of the improvement may be treatment effect of the services. The patients’ ratings of their satisfaction with the services were done by the patients, but in interviews done by the psychiatrists. This may have influenced the patients’ ratings of the CMHC services to be more positive, but still the satisfaction was highest with the GPs.

## Conclusions and implications

Patients with SMI in rural areas value local services that emphasize relationships and close collaborations among the CMHC, GPs and other primary care. Even in an area with a fairly comprehensive CMHC, the highest patient satisfaction was reported for GPs, indicating the potential key role of GPs for this patient group.

Clinical implications from our experiences and follow-up study are that GPs are important for patients with SMI, both as primary physicians and as collaborators with other primary care services and specialized mental health services. GPs are accessible close to where patient live and in less stigmatizing settings than specialized mental health services, they know the context in the local community, they know other local services as well as specialized mental health service and general hospital services, and they are the gateway to such services. GPs are also the main coordinators of the combination of somatic and mental health care. GPs often represent continuity of care, which is essential for building and maintaining a good personal relationship with the patient. The CMHC may give important comprehensive mental health services that are accessible to the patient, and the CMHC may support the GPs in their care for patients with SMI. Close collaboration between GPs, other primary care services and specialized mental health services like CMHCs is crucial for giving the comprehensive shared care that is needed.

Research implications from our experiences and study are that there is a need for more studies on how comprehensive care can be delivered in rural communities by collaboration among GPs, other primary care services and specialized mental health services. Patient experiences and recommendations should be studied in greater detail and with persons with service user experiences, caregivers and GPs involved in defining the research questions. Multi-center studies involving many rural areas should be done to have large enough samples and to learn from variations across sites.

Implications for service providers and policy makers are that they should support the aims for clinical practice and research described above. Further development of health services must both take into account the situation and needs in rural areas, as well as learn from experiences in rural areas by identifying and studying models and practices that seems function well.

## Ethics approval and consent to participate

The study was assessed by the Regional Committee on Medical Research Ethics in Health Region III as being a follow-up and quality assurance study of the patients of the CMHC and did not require approval (reg. no. 87/99-16.99). All patients included in the study gave written informed consent to participate on a form approved by the committee.

## Abbreviations

BPRS-E: Brief Psychiatric Rating Scale Extended version
CMHC: community mental health center
DSM-IV: Diagnostic and Statistical Manual of Mental Disorders, 4th Edition
GAF: Global Assessment of Functioning Scale
GP: general practitioner
HoNOS: Health of the Nation Outcome Scales
ICC: inter-rater reliability
OPCRIT: Operational Checklist for Psychotic Symptoms
PSF: Rating Scale for Practical and Social Functioning
SEPREP: Center for Psychotherapy and Psychosocial Rehabilitation of Psychoses
SMI: severe mental illness
SPSS: Statistical Package for the Social Sciences
