# Erratum to: Satisfaction with primary care and mental health care among individuals with severe mental illness in a rural area: a seven-year follow-up study of a clinical cohort

**DOI:** 10.1186/s13033-016-0072-8

**Published:** 2016-05-16

**Authors:** Torleif Ruud, Trond F. Aarre, Berit Boeskov, Per Ståle Husvåg, Rigmor Klepp, Synnøve Alet Kristiansen, Jorunn Sandvik

**Affiliations:** Division of Mental Health Services, Akershus University Hospital, 1478 Lørenskog, Norway; Institute of Clinical Medicine, University of Oslo, Oslo, Norway; Nordfjord Psychiatric Center, Nordfjordeid, Norway; Child and Adolescent Psychiatric Department, Center for Eating Disorders, Region Sjælland, Denmark; Child Welfare, Gloppen, Norway

## Erratum to: Int J Ment Health Syst (2016) 10:33 DOI 10.1186/s13033-016-0064-8

The original version of this article [1] unfortunately contained a mistake in the author list. The author Per Ståle Husvåg was incorrectly cited as “Per Sta Le Husvåg”. The updated author list has been provided in this erratum.

The original manuscript has been updated to reflect this change.
